# Barriers to transition to resource-oriented sanitation in rural Ethiopia

**DOI:** 10.1007/s11356-025-35887-6

**Published:** 2025-01-14

**Authors:** Thomas Ayalew Abebe, Josef Novotný, Jiří Hasman, Biruk Getachew Mamo, Gudina Terefe Tucho

**Affiliations:** 1 Department of Environmental Health Sciences and Technology, Institute of Health, Jimma University, Jimma, Ethiopia; 2 Department of Social Geography and Regional Development, Faculty of Science, Charles University, Prague, Czechia

**Keywords:** Resource-oriented sanitation, Eco-toilet, Ethiopia, Excreta recycling, Resource recovery, Willingness to pay

## Abstract

Recycling excreta resources through resource-oriented toilet systems (ROTS) holds transformative potential, yet adoption remains limited, especially where benefits could be high. This study aims to understand constraints hindering the adoption of ROTS in one such area in Ethiopia. Based on a survey among 476 households comprising 2393 individuals, we examine the plans to use ROTS and willingness to pay for ROTS and apply structural equation modelling to analyze the drivers of these two outcomes while comparing the explanative power of the extended technology acceptance model, extended theory of planned behaviour, and their combined model. While 40% of households expressed a plan to use ROTS and 20% reported willingness to pay for a subsidized ROTS with a biogas unit, merely 7% revealed both the plan to use and sufficient willingness to pay, highlighting the need to target both these complementary outcomes concurrently. The theory of planned behaviour showed the best explanative power, also revealing that these two outcomes are influenced by partly distinct sets of factors. Findings imply that common efforts to ease objective constraints through subsidies, which incentivize willingness to pay, are necessary but not sufficient for facilitating the adoption of ROTS. To simultaneously enhance intentions to use, it is also recommended to target psychosocial drivers, such as perceived behavioural control and perceived community support, through awareness creation, behaviour change activities, and community engagement techniques.

**Supplementary Information:**

The online version contains supplementary material available at 10.1007/s11356-025-35887-6.

## Introduction

The resource-oriented sanitation aims at recovering resources from human waste by sanitation technologies that are both safe for human health and environmentally friendly. An example is resource-oriented toilet systems (ROTSs) designed to close the nutrient loops between sanitation and agriculture, simultaneously saving both water and energy, particularly if connected to biogas units (Langergraber & Muellenger 2005; Hu et al. 2016). Therefore, ROTS can be seen as a niche innovation that can facilitate a sustainability transition to a new socio-technical regime (McConville et al. 2022). The gains might be especially high in countries like Ethiopia, examined in this study, where the continuing prevalence of unhygienic sanitation practices (Novotný & Mamo 2022; Abebe & Tucho 2020) coincides with the increasing pressures on land (Genet 2020; Ogato et al. 2021), frequent water scarcity, declining soil fertility, limited access to fertilizers (Gedamu 2020; Abay et al. 2022; Seyoum 2016), and energy poverty (Tucho et al. 2014; Mengistu et al. 2016).

Despite its transformative potential (Andersson et al. 2013; Guzha et al. 2005; Haq & Cambridge 2012), the adoption of ROTS has still been limited, particularly where the benefits could be high. Previous literature points at obstacles in various domains such as costs, political and institutional factors, supply chains, environmental conditions, the role of previous sanitation interventions, awareness, risk perception, or attitudes and norms around the acceptance (Simha & Ganesapillai 2017; Banamwana et al. 2022; Conroy & Mancl 2022; Gwara et al. 2021).

Facilitating sanitation change is known to be complex and context-dependent (Novotný et al. 2018; Chakraborty et al. 2022; Winter et al. 2018; McAlister et al. 2023; Novotný et al. 2024), and this holds even more for the adoption of ROTS that involves additional challenges around human excreta recycling and byproduct use. These aspects are also comparatively often studied (McConville et al. 2023; Banamwana et al. 2023; Gwara et al. 2021; Gwara et al. 2023; Williams et al. 2022a; Williams et al. 2022b), although the acceptance of excreta reuse is just one factor of the demand for ROTS adoption. However, there is considerably less research analyzing explicit intentions to use ROTS.

We also identified only two studies examining the willingness to pay (WTP) for ROTS, both conducted in high-income countries (Eom et al. 2021; Lamichhane & Babcock 2013). The absence of such research in low-income settings means another notable gap. Understanding users’ financial perspectives is crucial for informing policies that facilitate the transition to sustainable sanitation, alongside analyzing costs and benefits (Carrard et al. 2021). The unaffordability is a key issue even with regard to conventional sanitation in Ethiopia (Mamo et al., 2023a; b; Mamo & Novotný, 2024), and it has been recognized as a major barrier to ROTS uptake too (Conroy & Mancl 2022).

Moreover, the intersection between the intention to use ROTS and WTP for ROTS has not been studied at all. Intuitively, it may be assumed that they are overlapping measures of user demand. However, such an assumption can be misleading. These measures may, in fact, be unrelated and conceptually distinct, influenced by different sROTS of factors, and amenable to distinct interventions.

Alongside empirical gaps, theoretical understanding of the processes underlying the demand for ROTS is limited, constraining the ability to generalize empirical findings (Leviton 2017) and to transfer interventions across contexts (O'Cathain et al. 2019). The majority of previous research consists of exploratory case studies that did not engage with theories (e.g. Jensen et al. 2008; Andersson & Minoia 2018; Nawab et al. 2006; Dickin et al. 2018; Tumwebaze et al. 2011; Eelderink et al. 2017). Some studies into resource-oriented sanitation employed broader conceptual frameworks such as the socioecological system framework (Zhou et al. 2010; Trimmer et al. 2020; Machado et al. 2021) or the socio-technical transition framework (McConville et al. 2022), while the diffusion of innovation theory was utilized by two recent reviews (Banamwana et al. 2022; Conroy & Mancl 2022).

Regarding explicit analytical models, the theory of planned behaviour (TPB) proved useful for conceptualizing the main behavioural antecedents of human excreta recycling (Mariwah and Drangert 2011; Gwara et al. 2022). However, these studies did not examine how the TPB predictors (i.e. attitudes, social norms, and perceived behavioural control) specifically relate to behavioural intentions, omitting an important part of the TPB reasoning. Qualitative research by Banamwana et al. (2023) also documented the significance of the TPB predictors for the use of ROTS byproducts while additionally highlighting the role of perceived usefulness for the attitudinal shift in the perceptions of human excreta from waste to resource. It indicates the potential for integrating TPB with the technology acceptance model (TAM) because perceived usefulness is a central variable of the latter. This potential was explored by Ignacio et al. (2018) combining TAM and TPB in a single model (referred to as C-TAM-TPB) to explain the intention to use ROTS in the Philippines. Although tempting, combining distinct theories into the C-TAM-TPB presents challenges (Cheng 2019). An unresolved question remains: Would opting for a single theory, such as TPB or TAM, be more parsimonious?

This article addresses the empirical and theoretical gaps outlined above. Our general goal is to understand the demand for the adoption of ROTS in rural Ethiopia. Based on data collected in 476 farming households comprising 2393 individuals in the Girar Jarso District, Oromia, we combine an exploratory network analysis with confirmatory structural equation modelling to examine the outcome measures of user demand, namely, the intention to use ROTS and WTP for ROTS and their predictors. Our study has the following objectives:

- To descriptively characterize the potential for excreta recycling and user demand for ROTS
- To explore the binary relationships between two outcome variables of user demand (intention to use ROTS and WTP for ROTS) and their predictors using network analysis
- To compare the power of TAM, TPB, and C-TAM-TPB for explaining variance in the two outcomes using structural equation modelling
- To investigate by structural equation modelling how specific predictors derived from TPB and TAM (outlined in the next section) influence the outcomes
- To examine the role of predictors that are external to TPB and TAM

This article adds to the understanding of social determinants of environmental health. It represents the first attempt to quantitatively analyze the demand for ROTS in rural Ethiopia, where ongoing efforts are being made to facilitate its adoption through specific interventions. It is also the first study to simultaneously analyze the intention to use ROTS and WTP for ROTS, demonstrating their complementarity. Moreover, the study offers a valuable theoretical contribution by testing the applicability of well-known theories in understanding the demand for ROTS.

## Methods

### Conceptual model and analytical strategy

**Fig. 1:**
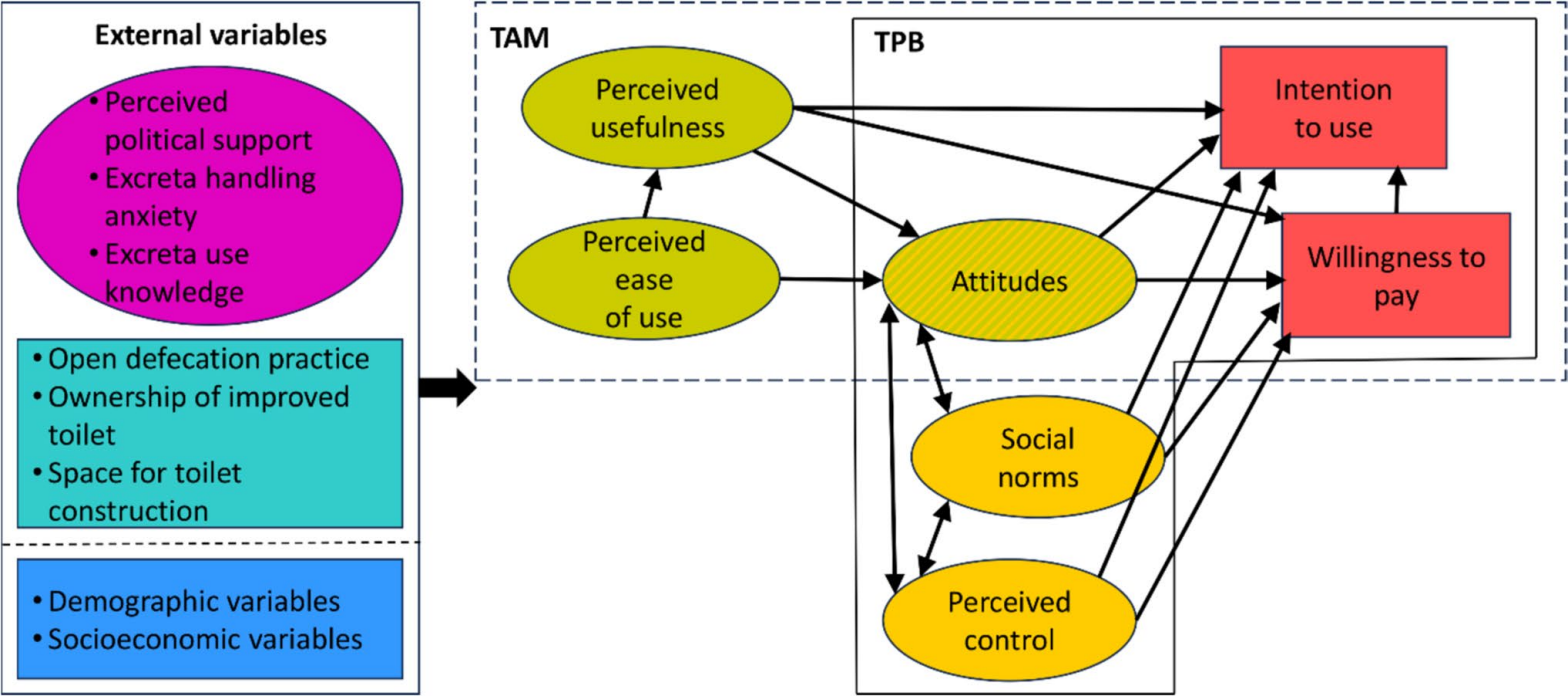
Models employed in this study: TAM, TPB, and their combination (C-TAM-TPB). Note: Rounded boxes indicate variables that are measured as latent constructs

Both TPB and TAM view behavioural intention (intention to use or intention to pay) as the primary predictor of actual behaviour. The TAM (Fig. 1, area indicated by a dashed line) anticipates that the intention to adopt a technology can be explained by attitudes towards the technology determined by its perceived ease of use and its perceived usefulness. Additionally, it acknowledges that perceived ease of use can positively influence perceived usefulness and that there may be a direct effect of the perception of usefulness on intention to adopt (Davis 1985). The TPB (Fig. 1, a solid line on the right side), a more general model, posits that the intention can be explained by three psychosocial constructs: attitudes towards the behaviour, perceived social norms, and perceived behavioural control (Ajzen 1991).

We additionally explore the joint TAM and TPB model referred to as C-TAM-TPB adapted from Ignacio et al. (2018). We nevertheless acknowledge that combining two distinct theories into a single model increases the model complexity, may lead to potential information redundancy, and poses the risk of unstable relationships (Cheng 2019). Therefore, one of our objectives is to compare the performance of the C-TAM-TPB model with specifications derived from TPB and TAM separately.

General psychological theories like TPB or TAM (and thus also C-TAM-TPB) allow for extensions by integrating external variables whose effects are expected to be channelled through the core TPB and TAM constructs. Consideration of these external variables (throughout this paper, variables other than the core TPB or TAM constructs are referred to as external variables) is important for shedding light on the underlying mechanisms and the contextual influencers. As indicated on the left side of Fig. 1, three sets of external variables are analyzed in this study. The first includes the constructs measuring perceived political support for ROTS adoption, knowledge about the potential of excreta recycling, and perceived anxiety associated with excreta handling. The second group comprises three measures of the current sanitation conditions, and the third set covers the selected demographic and socioeconomic characteristics.

A salient feature of this study is that it examines simultaneously two outcome measures of user demand. Based on previous literature from different thematic areas, a positive correlation between intention to use and WTP might be anticipated, though the direction of this relationship is open to discussion (Sanchez-Garcia et al. 2021; Park et al. 2022; Irfan et al. 2020; Schniederjans et al. 2014). In low-income settings, it is reasonable to presume that WTP influences the intention to adopt rather than the reverse, and we specify this path in Fig. 1 accordingly. Recent research conducted in South Ethiopia supports this assumption, indicating that the WTP for hygienic sanitation products positively influences the intention to upgrade toilets (Mamo et al. 2023a).

Aligning with the specific objectives proposed in the introduction, our analytical strategy consists of the following steps:

- In Step 1, we provide a descriptive characterization of the excreta recycling potential and user demand for ROTS.
- In Step 2, we use network analysis to examine the patterns of binary relationships between variables considered in our conceptual model.
- In Step 3, we apply structural equation modelling to sequentially examine the performance of TAM, TPB, and C-TAM-TPB.
- In Step 4, we interpret the role of the individual TAM and TPB predictors.
- In Step 5, we use the best-performing model from Step 3 to compare the indirect effects of external variables with their direct effects.

### Study area

The Girar Jarso District is located in the North Shewa Zone, Oromia Region, Central Ethiopia. As in the majority of rural Ethiopia, the primary livelihood for the local population involves mixed agriculture, predominantly focused on cereal and pulse crop production alongside livestock grazing (Abi et al. 2020). The district was purposely selected for our household survey, based on familiarity with the situation, including efforts to facilitate the adoption of ROTS by a subsidy-based programme promoting domestic biodigesters with toilets. According to the district water and energy office, less than one percent of the districts’ households had biogas units at the time of our survey.

### Household survey

The survey was conducted from September to October 2022 through structured interviews and direct observations in a district-representative sample of 636 households. For this study, we used a subsample of households who have land for farming (*N* = 476) and could thus directly utilize humanure. The seven kebeles (the smallest administrative units) were selected randomly for the list of 17 kebeles of the Girar Jarso District. A random walk technique was used to sample households within the kebeles reflecting their size and spatial structure. Heads of households were interviewed, and in the absence of heads of households, the adult members of the household (preferably spouses) were interviewed. Twelve trained data enumerators, proficient in the Affan Oromo, language spoken in the study area, collected data under the supervision of two supervisors.

The survey instrument (Supplementary Information 1) consisted of 122 questions of which 22 assessed parameters related to sanitation facilities and their surroundings. In addition to other sections, it included a set of statements to measure the theoretical constructs derived from TAM and TPB that were mostly adopted by Ignacio et al. (2018).

### Measures

The first outcome, intention to use ROTS, was assessed by gauging agreement with the statement “I plan to use the ROTS in the next 12 months”, measured on a 5-point scale with the lowest and highest category corresponding to the strong disagreement and strong agreement, respectively. The second outcome, WTP for ROTS, was determined through a contingent valuation procedure. Respondents were initially informed about two ROTS technology options available in the surveyed area and their respective costs. The first option involved a double vault urine-diverting dry toilet, with costs ranging from 12,000 to 25,000 ETB, depending on the type and extent of utilization of locally available materials (estimates derived from consultations with three local experts). The second option, a biogas toilet, had costs ranging from 25,000 to 50,000, contingent on the digester’s volume. The participants were also informed about the possibility of utilizing a government subsidy. Under this option, households have to contribute 15,000 ETB for a 10 m^3^ biogas digester. Respondents were then presented with an initial bid of 50,000 ETB for ROTS. Depending on their response, subsequent bids increased if they agreed or decreased if they disagreed. This approach allowed for the establishment of a continuous WTP variable. For the analysis, the WTP variable was further adjusted using a square root transformation to reduce skewness.

Composite reliability was evaluated for all latent constructs depicted in Fig. 1, and two items were excluded due to the low factor loadings. Items eventually used for the construction of these constructs can be found in Supplementary Information 2 (Table S1).

Three dichotomous variables capturing current sanitation conditions were used. The first evaluates the presence of available space for toilet construction. The second gauges ownership of an improved latrine, defined as a functional sanitation facility with a solid slab platform, not shared with other households. The third assesses the presence of open defecation practices in households. A household was classified as practicing open defecation if it lacked a functional sanitation facility and/or if apparent signs of open defecation were recorded in the observation.

We also consider various demographic and socioeconomic variables including the age, sex, and education of the household head, household size, income (annual, both in-cash and in-kind, from various sources), livestock ownership (measured in tropical livestock units), farmland size, and a media sources availability index (derived from the ownership of mobile phones, TVs, radios).

### Ethics

An informed verbal consent was sought and obtained from each study participant. All participants took part in our field research voluntarily, and they were assured of the anonymity and confidentiality of their personal information. They were also informed about a right to withdraw from the survey at any time. Prior to the survey, our research received formal approvals from local authorities. Ethical clearance was obtained from the Institutional Review Board of Jimma University (No. JUIH/IRB/12/22).

### Data analysis

To explore the patterns of binary relationships between variables (Step 2), we calculated pairwise correlation coefficients for all variables using SPSS AMOS 28 (Arbuckle 2019). We then considered the statistically significant correlations (*p* < 0.01) and used a weighted force-directed algorithm to visualize the network in Cytoscape (Shannon et al. 2003).

In Steps 3 and 4, we estimated structural equation models using SPSS AMOS 28. Initially, we modelled the TAM, TPB, and C-TAM-TPB configurations separately, excluding demographic and socioeconomic variables. Based on modification indices, we made slight adjustments to the models by adding a few direct paths between external variables and outcomes. In Step 5, the best-performing model was then re-estimated while incorporating demographic and socioeconomic variables as external factors.

## Results

### Descriptive findings

The descriptive characteristics of the sample are in Supplementary Information 2 (Table S2).

#### Sanitation situation

Nearly one-fourth (23%) of the households lacked any sanitation facility. Fifty-five percent (55%) had unimproved pit latrines, and 21% had improved pit latrines. Apparent signs of open defecation practices were recorded in 53% of households, while more than half of respondents (51%) from latrine-owning households admitted that they do not use their latrines consistently. Despite this, 91% of them expressed satisfaction with their sanitation facilities, and the shares of satisfied were high among both those with improved latrines (98% of satisfied) and unimproved facilities (89%).

The majority of the households in our sample did not have waste conversion facilities: only two families had biogas digesters, and two other households utilized composting toilets. In terms of sludge management, 93% of toilet owners mentioned covering the full pit with soil and digging another one, only 4% of families with toilets reported composting, while the remaining 3% empty the pits and release the sludge elsewhere as waste.

#### Potential for agricultural reuse of humanure

The majority of 97% and 85% of households regularly employ inorganic fertilizers and animal manure, respectively. A majority of them also reported shortages of fertilizers, with 67% stating this for inorganic fertilizers and 71% for animal manure. While 65% stated that they would use humanure if properly sanitized and 61% stated that they would consume crops cultivated with the use of humanure, only 17% reported using humanure for crop cultivation, and 64% opposed handling faecal material in any way. The most common barrier was smell (40%), followed by concerns about possible health risks (15%).

#### Demand for ROTS

The majority of respondents (68%) acknowledged the potential economic benefits of ROTS, and 64% believed in its possible positive health impacts. However, only 10% reported a strong intention to adopt ROTS within a year, 30% moderately agreed with such a plan, and 13% were neutral or undecided. The most frequently cited motivation for ROTS adoption was nutrient recovery (33%), followed by efforts to improve hygiene (16%) and health (6%).

**Fig. 2:**
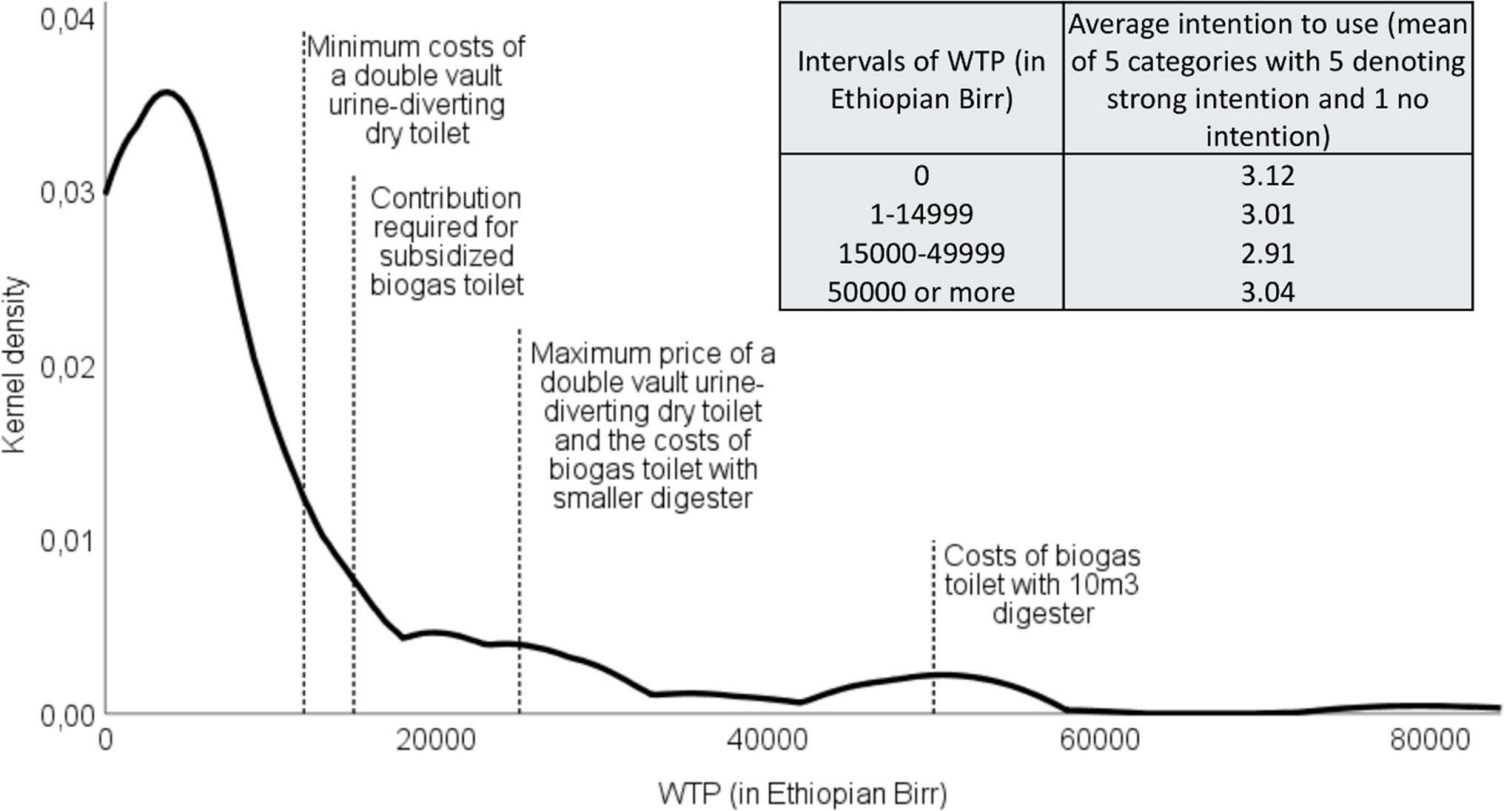
Kernel density plot for WTP for ROTS and mean intention to use ROTS by intervals of WTP

The distribution of WTP for ROTS and its relationship to the reported intention to use is shown in Fig. 2. Only 22% of respondents would pay 12,000 ETB or more, the lower limit for the estimated price of a double vault urine-diverting toilet; 20% reported WTP corresponding to at least 15,000 ETB, which is the required household contribution for obtaining a subsidized biogas toilet; and only 6% would be willing to cover the full cost of a biogas toilet with a 10m^3^ digester, amounting to 50,000 ETB. Costs were also perceived as the most significant barrier to ROTS adoption (reported by 72%), along with the perception that ROTS is too complicated (14%).

A notable finding is the limited overlap between the two central outcome measures. They were statistically unrelated, and an inserted table in Fig. 2 shows that the reported plan to use ROTS was on average strongest for those unwilling to play. Only one-third of those who reported sufficient WTP to cover the subsidized biogas toilet simultaneously reported the intention to use ROTS. Across the entire sample, only 7% revealed both the intention to use ROTS and sufficient WTP.

### Measurement model

Results obtained from the confirmatory factor analyses are reported in Table 1. The internal consistency was acceptable for all latent constructs, except perceived ease of use and excreta use knowledge. We did not find better options and decided to keep these two constructs despite their lower internal consistency, also by considering the overall model fit indicators that were acceptable. The lesser consistency of items used for the two constructs was further indicated by their moderate factor loadings. However, even the minimum loading of 0.441 can be accepted. The loadings were stable across the examined model specifications. These results document that the considered items provide relevant information for measuring their pertaining latent constructs. The fit indices in Table 1 also suggest the measurement adequacy for all three models.

**Table 1:** Measurement model for structural equation modelling: factor loadings for particular items and models (columns on the right side), Cronbach alphas (left column), and model fit indices (bottom rows)

Latent variable	Statements used for particular items	TAM	TPB	C-TAM-TPB
Perceived usefulness (Cα = 0.788)	I found ROTS useful and efficient in improving sanitation of my community	0.846	–	0.777
	Using the ROTS would motivate me to improve sanitation in my community	0.769	–	0.838
Perceived ease of use (Cα = 0.513)	My interaction with the ROTS would be clear and understandable	0.565	–	0.549
	It is easy for me to learn and become skilful at using the ROTS	0.610	–	0.601
Attitudes (Cα = 0.834)	Using the ROTS is a good idea	0.686	0.762	0.687
	Using the ROTS can be a source of income generation	0.774	0.702	0.777
	The ROTS makes my lifestyle more interesting	0.767	0.836	0.764
	I like to use the ROTS	0.724	0.646	0.717
Social norms (Cα = 0.836)	People who are important to me would think that using ROTS is good for me	–	0.901	0.891
	People who influence my behaviour would think that I should use ROTS	–	0.664	0.676
	In general, my community has supported use of ROTS	–	0.798	0.800
Perceived control (Cα = 0.701)	I have resource necessary to use ROTS (money, space, etc.)	–	0.632	0.629
	I know the benefit of using the ROTS	–	0.743	0.758
	A specific body is available for assistance in case of difficulties using ROTS	–	0.623	0.621
Perceived political support (Cα = 0.932)	Community leaders will support implementation, operation, and maintenance of ROTS	0.917	0.913	0.912
	The local government will support implementation, operation, and maintenance of ROTS	0.951	0.956	0.956
Excreta use knowledge (Cα = 0.591)	Human excreta are resource for soil	0.562	0.543	0.554
	Sanitized human excreta can be used as fertilizer	0.770	0.794	0.778
	Crops fertilized with human excreta are good for consumption	0.444	0.441	0.445
Excreta handling anxiety (Cα = 0.869)	Handling excreta is great health risk	0.596	0.596	0.595
	Human excreta should not be handled in any way	0.642	0.647	0.642
	Human urine has no benefit to humans	0.467	0.464	0.467
	It is taboo to handle urine	0.796	0.794	0.795
	Human faeces have no benefit to humans	0.555	0.553	0.554
	It is taboo to touch faeces	0.849	0.847	0.851
	It is taboo to touch treated faeces	0.849	0.851	0.848
Model fit indices	Incremental Fit Index (IFI)	0.940	0.921	0.906
	Tucker-Lewis Index (TLI)	0.924	0.902	0.883
	Comparative Fit Index (CFI)	0.939	0.920	0.905
	Root mean square error of approximation (RMSEA)	0.060	0.069	0.068
	Chi-square to degrees of freedom ratio (χ^2^/df)	2.737	3.259	3.198

Notes: except for excreta use knowledge, all statements were measured on a 5-point scale that ranged from strong and moderate disagreements to moderate and strong agreements with a middle category for neutral or undecided. Statements ascertaining the excreta use knowledge were measured on a 3-point scale

*Cα* Cronbach alpha.

### Exploratory network analysis of bivariate relationships

**Fig. 3:**
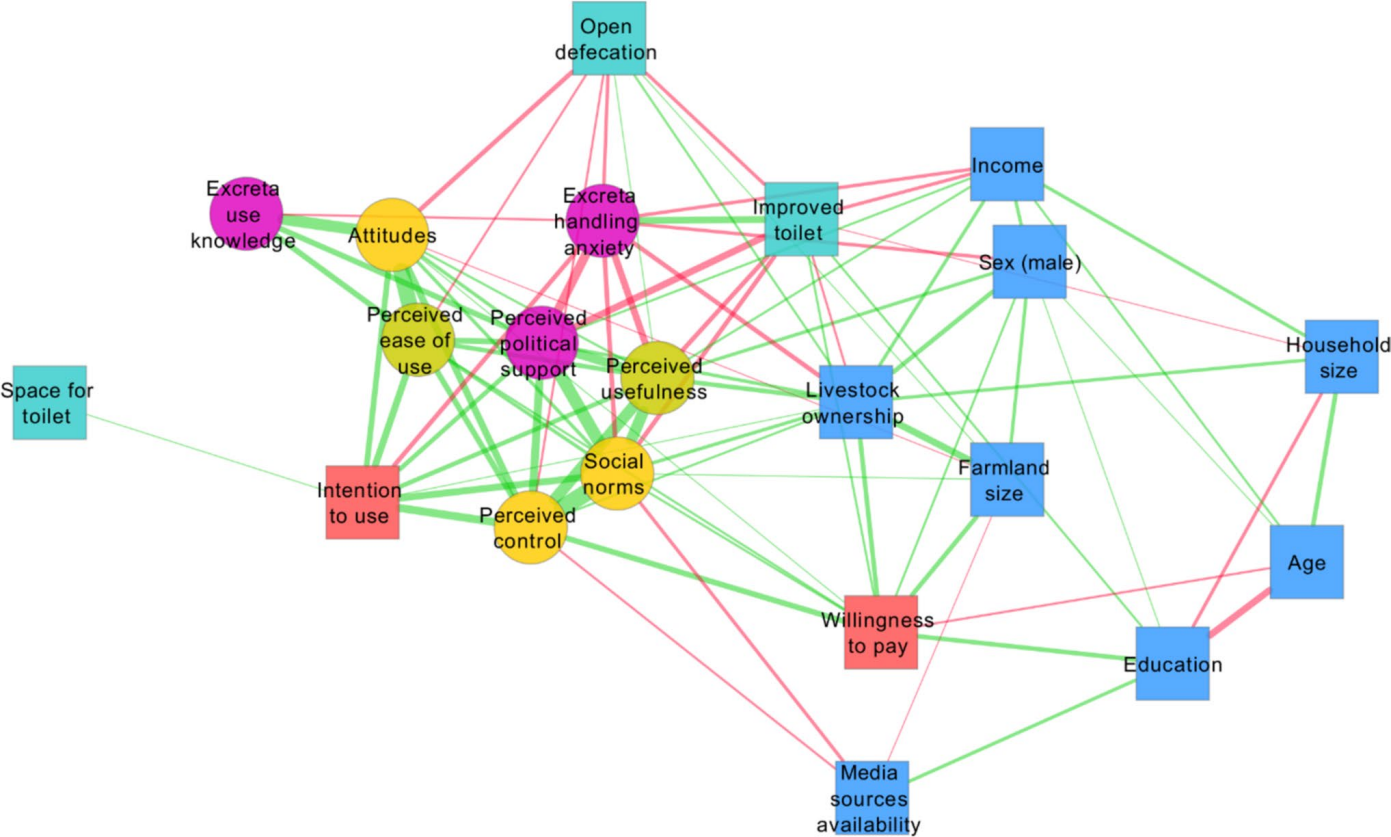
Network of binary relationships. Notes: the width of edges is proportional to the strength of relationships. All displayed relationships were statistically significant with the *p*-level below 0.01. Positive relationships are denoted by green edges, while negative relationships are represented in red. Node shapes and colours distinguish the types of variables based on their roles in the conceptual model in Fig. 1

Network visualization presented in Fig. 3 offers valuable insights into the aggregate pattern of the binary relationships. All latent constructs (round-shaped nodes) cluster near the intention to use ROTS, confirming their relevance as the predictors of this outcome. In contrast, WTP is relatively less connected to this cluster, though it exhibits statistically significant correlations with the TPB predictors (particularly perceived behavioural control) and with the perceived ease of use. It is positioned closer to certain socioeconomic (education, livestock, and land ownership) and demographic (age and sex) variables. Of the socioeconomic variables, the ownership of livestock revealed a central position, with positive associations with the majority of the ROTS-related measures, including the two outcomes.

The results highlighted the potentially consequential role of the sanitation-related variables. The availability of space for toilet construction was positively associated with the intention to use but not with other variables which is intuitive. Having an improved latrine was associated with a comparatively lower perception of the usefulness of ROTS, lower perceived political support, lower perception of social norms around ROTS, and higher excreta handling anxiety while practicing open defecation correlated with the weaker attitudes towards ROTS, lower perceived ease of use, lower behavioural control, and lower excreta handling anxiety, but higher perception of usefulness of ROTS.

### Structural equation models

For building three structural equation models informed by TAM, TPB, and C-TAM-TPB, we considered their specifications as in Fig. 1, including external variables, except demographic and socioeconomic ones. Based on the modification indices, we additionally included three direct paths (direct effects of space for toilet construction and excreta handling anxiety on intention to use and improved toilet ownership on WTP). These added paths aligned with the results obtained in the network analysis above. Furthermore, a path between social norms and WTP was excluded due to the multicollinearity arising from a high correlation between social norms and perceived behavioural control.

**Fig. 4:**
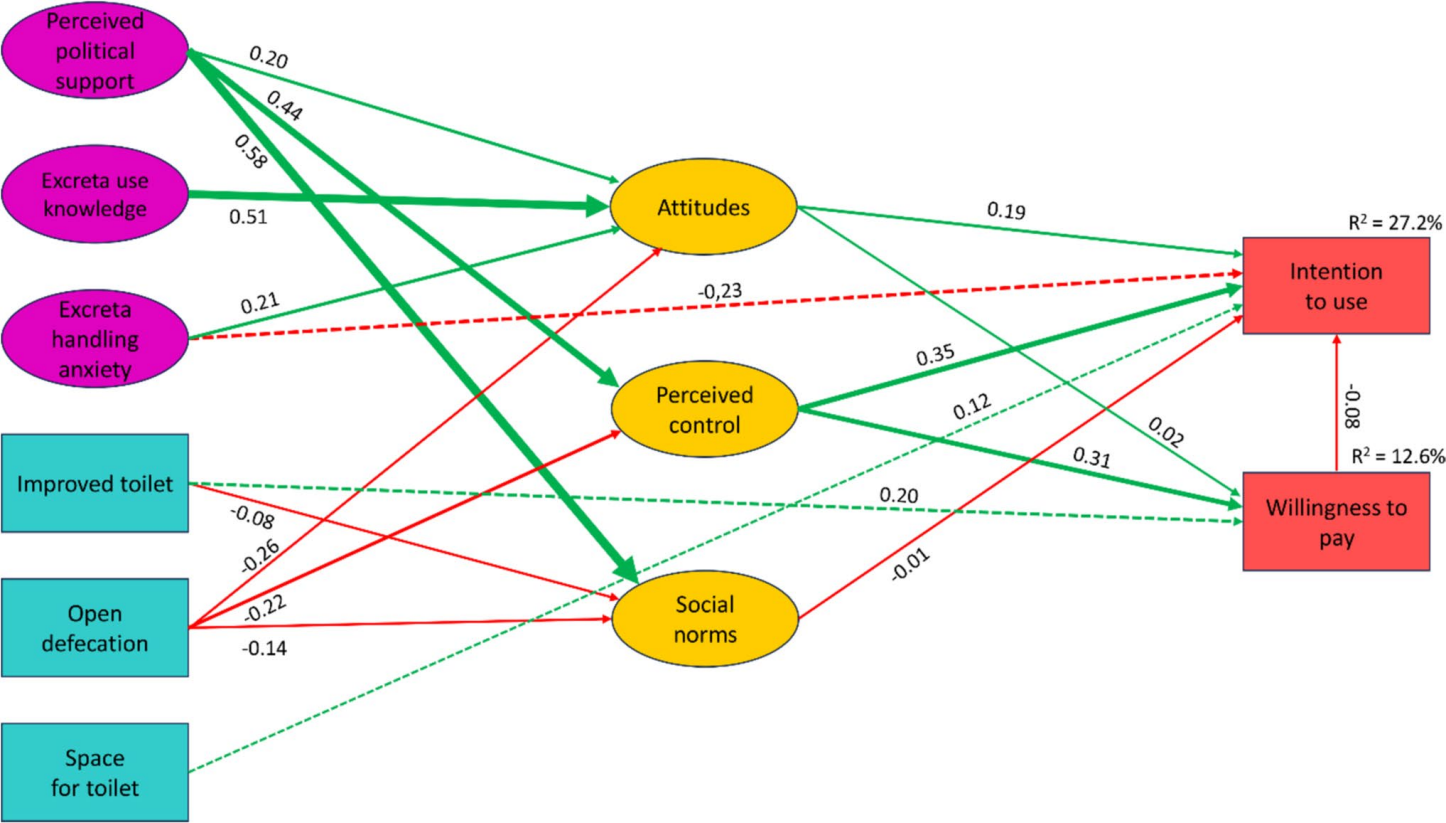
Estimates for the TPB-informed structural model. Notes: only statistically significant paths (*p* < 0.01) were considered for external variables on the left side

The goodness-of-fit indicators were acceptable for all three models (Table 2). The slightly better goodness-of-fit of the TAM specification was primarily attributed to its higher measurement adequacy rather than superior performance in modelling structural relationships. The TPB and C-TAM-TPB explained the higher proportions of variance in both outcomes. Since TPB provides more general theoretical guidance and is also more parsimonious, we decided to prioritize it as the best-performing model presented in Fig. 4, with Table 3 showing the estimated direct, indirect, and total effects of individual predictors. Supplementary Information 2 (Figs. S1 and S2, Tables S3 and S4) provides results for the TAM and C-TAM-TPB specifications.

**Table 2:** Fit indices for structural equation models and their explained variance in outcomes

Indices		TAM	TPB	C-TAM-TPB
Structural equation models fit indices	IFI	0.919	0.900	0.877
	TLI	0.902	0.880	0.853
	CFI	0.919	0.899	0.876
	RMSEA	0.057	0.065	0.066
	*χ*^2^/df	2.548	2.987	3.088
Proportion of explained variance	*R*^2^ for intention to use ROTS	0.22	0.27	0.27
	*R*^2^ for WTP	0.07	0.13	0.12

**Table 3:** Standardized direct and indirect effects of variables in the TPB specification considered in Fig. 4

Variables	Effects on intention to use ROTS			Effects on WTP for ROTS		
	Direct	Indirect	Total	Direct	Indirect	Total
Attitudes	0.194	− 0.001	0.193	0.016	–	0.016
Perceived control	0.349	− 0.026	0.323	0.306	–	0.306
Social norms	− 0.013	–	− 0.013	–	–	–
Perceived political support	–	0.174	0.174	–	0.138	0.138
Excreta use knowledge	–	0.098	0.098	–	0.008	0.008
Excreta handling anxiety	− 0.232	0.041	− 0.191	–	0.003	0.003
Improved toilet	–	− 0.016	− 0.016	0.205	–	0.205
Open defecation	–	− 0.119	− 0.119	–	− 0.071	− 0.071
Space for toilet	0.117	–	0.117	–	–	–

The results uncovered only a very weak relationship between the WTP and intention to use. The variance in the latter outcome was considerably better explained by the final model. This is consistent with findings obtained in the exploratory network analysis and the theory. Perceived behavioural control was clearly the strongest predictor of both intentions to use and WTP. Attitudes towards ROTS revealed a notable relationship with intentions to use, while perceived social norms were almost unrelated to the examined outcomes.

An expected direct negative effect of excreta handling anxiety on intention to use was revealed. Similarly, the availability of space for toilet construction affected the intention to use ROTS directly without shaping the considered intermediate TPB predictors.

**Fig. 5:**
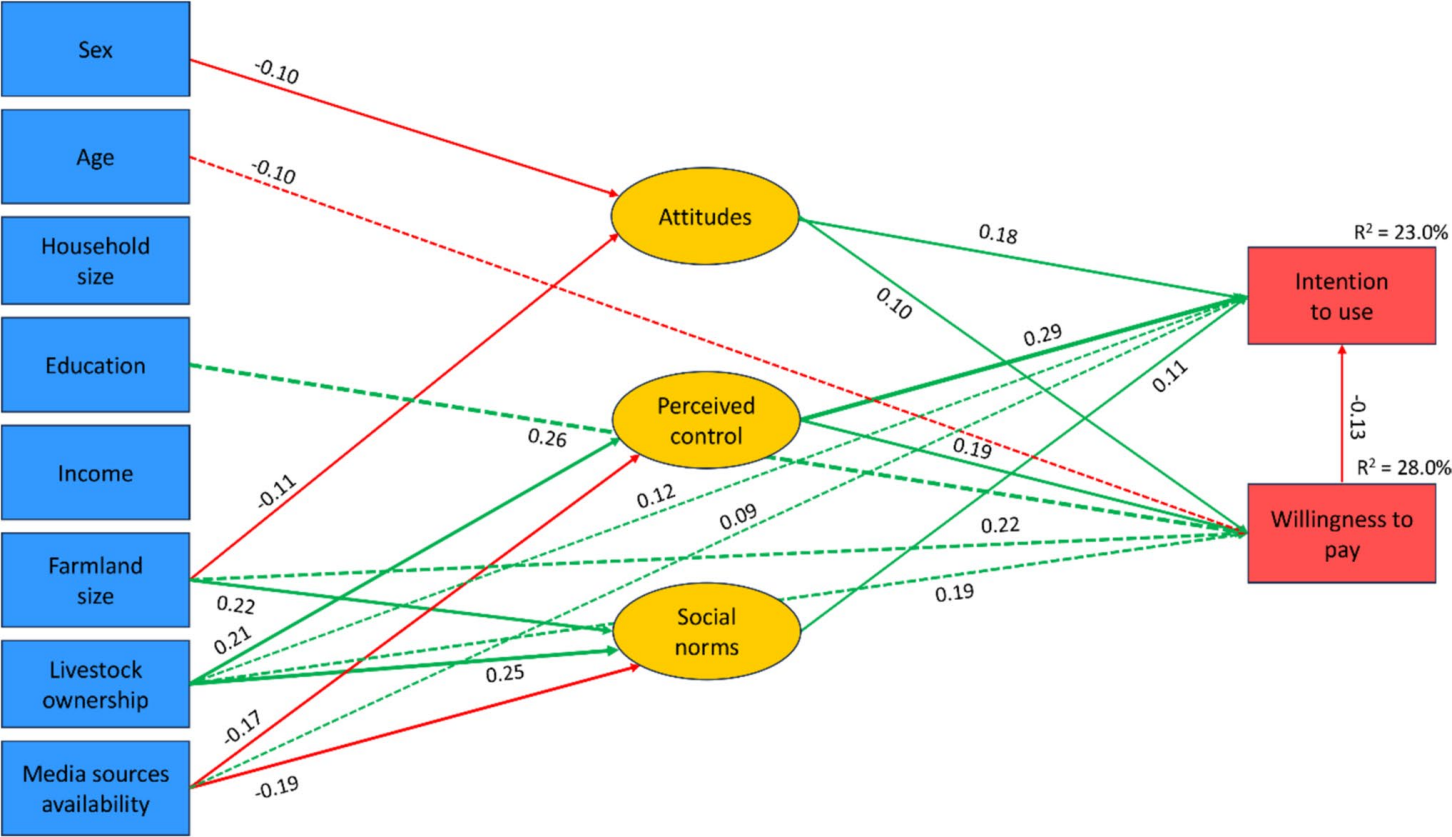
Estimates for the TPB specification with demographic and socioeconomic external variables

In the final step, we estimated a TPB-informed model that incorporated demographic and socioeconomic characteristics (Fig. 5; quantification of effects is in Table 4). The fit indices in terms of IFI, TLI, CFI, RMSEA, and χ^2^/df corresponded to 0.937, 0.911, 0.935, 0.052, and 2.297, respectively. This indicates a better fit than for previous models. Compared to the previous model in Fig. 4, R^2^ decreased for intention to use from 27.2 to 23.0%, but it increased notably for WTP from 12.6 to 28.0%. This is consistent with the pattern uncovered by the network visualization in Fig. 3.

**Table 4:** Direct and indirect effects of variables in TPB specification with demographic and socioeconomic external variables (as in Fig. 5)

	Effects on intention to use ROTS			Effects on WTP for ROTS		
	Direct	Indirect	Total	Direct	Indirect	Total
Attitudes	0.177	− 0.013	0.164	0.096	–	0.096
Perceived control	0.293	− 0.025	0.268	0.191	–	0.191
Social norms	0.110	–	0.110	–	–	–
Sex	–	− 0.016	− 0.016	–	− 0.009	− 0.009
Age	–	0.013	0.013	− 0.099	–	− 0.099
Household size	–	–	–	–	–	–
Education	–	− 0.034	− 0.034	0.261	–	0.261
Income	–	–	–	–	–	–
Farmland size	–	− 0.046	− 0.046	0.219	− 0.010	0.209
Livestock ownership	0.116	0.059	0.175	0.188	0.040	0.228
Media sources availability	0.092	− 0.067	0.025	–	− 0.033	− 0.033

The sex and age of the household head, household size, and income exhibited minimal or no impact on both outcome variables, as well as on the proximate TPB predictors that tend to be weak mediators of the effects of socioeconomic and demographic factors. The most notable positive direct effects on WTP were identified for education and the size of land ownership, while livestock ownership was positively related to both outcomes.

## Concluding discussion

The potential of resource-oriented sanitation to address the multiple challenges of unhygienic sanitation, environmental degradation, agricultural productivity, and energy production simultaneously is high in Ethiopia but largely untapped thus far. This article presents one of the first studies examining the user demand for ROTS, focusing on a region where these technologies have been promoted but still remain very rare.

Our joint focus on both the intention to use ROTS and WTP proved valuable, revealing that these outcomes were statistically unrelated yet thematically complementary. Interestingly, those households with higher WTP for ROTS showed slightly lower intentions to use ROTS. The intention to use ROTS was primarily influenced by psychosocial factors, while WTP was more tied to sanitation-related and socioeconomic characteristics, reflecting objective constraints on the capacity to invest in ROTS. We thus demonstrated that the two outcomes are conceptually distinct, contesting studies that comprehend behavioural intentions and WTP as interchangeable (e.g. Sanchez-Garcia et al. 2021) or unidirectionally conditional upon each other (e.g. Park et al. 2022; Irfan et al. 2020; Schniederjans et al. 2014).

Previous literature argued that financial and material incentives are needed to improve the sanitation situation in Ethiopia (Gebremariam & Tsehaye 2019; Afework et al. 2022; Mamo et al. 2023a, b). This need becomes even more critical for the ROTS, which are more expensive than pit latrines common across Ethiopia, while further targeted support may also be required to ensure sustainability (Trimmer et al. 2022). In this study, 40% of households expressed at least moderate intention to use ROTS, but only about one-fifth of households indicated a WTP sufficient to cover either the minimum cost of the dry urine-diversion eco-toilet or the cost of a subsidized biogas toilet. This means that the current subsidy offered to households in the study area may be sufficient for a smaller group of early adopters. However, this support is limited to biogas digesters, which pose their own issues and may primarily serve purposes other than human faecal waste management (Roubík et al. 2016; Balgah et al. 2023; Laré et al. 2024). Furthermore, we observed that high WTP does not automatically align with a strong intention to use. Only 7% of respondents reported both the intention to use ROTS and WTP sufficient for adopting a subsidized ROTS. Our findings can thus assist in more effectively targeting support towards households with characteristics instrumental to the actual demand for ROTS.

The ownership of livestock and farmland, as well as attained education, emerged as significant predictors of WTP confirming the influence of households’ socioeconomics. Additionally, the roles of livestock and land ownership are likely to reflect the need for sufficient inputs for biogas digesters and opportunities for humanure utilization.

Challenges related to the adoption of ROTS are complex and extend beyond economic costs. One such complexity involves the role of current sanitation conditions. We found that households who already have improved toilets and consistently use them (15% of the sample) exhibited both comparatively higher WTP and intention to use. Practicing open defecation (prevalent in the study area) is associated with the disbelief in own’s capacity or ability to adopt and use ROTS and weaker intentions to use ROTS. These two observations imply that the subgroup with no or unimproved latrines that are inconsistently used (46% of the sample) represents the least favourable subgroup regarding potential ROTS adoption.

Interestingly, households with improved latrines were, on average, poorer families than the rest of the sample but had better-educated household heads. The attained education also emerged as a significant direct predictor of WTP for ROTS. These findings thus confirm the positive role of education in enhancing the willingness to invest in better sanitation. Unlike general education, specific knowledge about the potential of human excreta reuse revealed a modest indirect effect on the intention to use ROTS by enhancing perceived behavioural control.

Our analysis consisted of the descriptive, exploratory, and confirmatory parts, of which each was valuable. The descriptive part uncovered generally positive views on the reuse of human excreta and the consumption of cultivated crops, although these perceptions were far from uniform. The exploratory network analysis then uncovered tight interlinkages within the cluster of considered psychosocial constructs and distinct positions of the observed contextual sanitation and socioeconomic characteristics.

In the confirmatory analyses, we found that all three theoretical models, informed respectively by TAM, TPB, and C-TAM-TPB and extended by the same set of relevant external variables, could be employed for modelling the intention to use ROTS and WTP. However, the TPB specification was identified as the best performing when considering the explained variance, parsimony, and generality. The results obtained based on the TAM are nevertheless also notable, specifically the significant positive effects of the perceived ease of use on both outcomes. TPB subsumes this perceived ease use into a broader notion of perceived behavioural control. At the same time, we showed that this construct is not only important per se, but also because of mediating the effects of external variables such as the perception of political support, the practice of open defecation (negative effects), and livestock ownership.

Our results have some straightforward implications. Both sufficient intentions to use and WTP are necessary preconditions for the adoption of ROTS. Current efforts for easing objective constraints through subsidies incentivize WTP but may not be sufficient to enhance intentions to use. To influence the latter, psychosocial drivers should be targeted by an appropriate campaign. It should address awareness and perceptions related to the excreta handling anxiety, knowledge about its recycling potential, perceived difficulties of ROTS use, its complexity, user-unfriendliness, inaccessibility, unaffordability, unattainable inputs, or missing skills and competences. Our results also underscore the importance of perceived political support, including the support from local government officers and, primarily, from community leaders and concerning grassroot-level installation, operation, and maintenance of ROTS.

Our study identified the demand-side challenges of the ROTS adoption in rural Ethiopia. Our findings may be useful for guiding interventions to target households with the highest likelihood of ROTS adoption, hopefully facilitating success stories that can serve as powerful motivators for other families. However, achieving the latter is unlikely through specific interventions alone and contingent upon the improvement of the general socioeconomic situation.

## Supplementary Information

Below is the link to the electronic supplementary material.

Supplementary Information 1 (PDF 272 KB)

Supplementary Information 2 (DOCX 403 KB)

## Data Availability

The data used in this study are available from the corresponding author upon reasonable request.
