# Supplementary material for: Barriers to transition to resource-oriented sanitation in rural Ethiopia
Source: Environ Sci Pollut Res Int. 2025 Jan 14;32(5):2668–81. doi: 10.1007/s11356-025-35887-6 (PMC11802593; doi:10.1007/s11356-025-35887-6)
Supplement: Supplementary file 1 — Supplementary Information 1 (PDF 272 KB) [file 11356_2025_35887_MOESM1_ESM.pdf]

PART- *A: **SOCIO-DEMOGRAPHIC CHARACTERISTICS***

| PART A: HOUSEHOLD QUESTIONNAIRE | | | |
|---|---|---|---|
| SR.No. | Questions | Coding Categories | Skip |
| 101 | Sex of the head of the household | 1. Male<br>2. Female | |
| 102 | Age of head of the household | _____________years | |
| 103 | Occupation of the head of the household | 1. Farmer<br>2. Craftsmen<br>3. Casual labour (short term employment…)<br>4. Self-employment (own enterprise, incl. petty trade)<br>5. Merchant<br>6. Government Employee<br>7. Housewife<br>8. Pensioner (retired)<br>9. Others: ____________ | |
| 104 | Educational status of head of the household | 1. Illiterate<br>2. Write and read<br>3. Primary school<br>4. Secondary school<br>5. College and above | |
| 105 | The ethnicity of the head household | 1. Oromo<br>2. Amhara<br>3. Tigre<br>4. Gurage<br>5. Silte<br>6. Other ____________ | |
| 106 | The religion of the head of household | 1. Orthodox<br>2. Protestant<br>3. Muslim<br>4. Catholic<br>5. Others ______________ | |
| 107 | Family size in household | ____________in number | |
| 108 | What is average annual household income?<br><br>NB: first all sources of income (both in cash and in kind income) for the household will be identified then the enumerator estimates the annual income in cash (main sources of income could be: from livestock sale, livestock products, from craft sale, from harvesting or other type of activities which are source of income) | ____________in ETB | |

| | | | |
|---|---|---|---|
| 109 | Housing occupancy status/ Homeownership | 1. Rent<br>2. Own<br>3. Other ___________ | |
| 110 | Having children aged < 10 years | _______________in numbers | |
| 111 | Having people aged > 60 years | ________________in numbers | |
| 112 | Marital status of head of household | 1. Married<br>2. Single<br>3. Widowed<br>4. Divorced<br>5. Separated | |
| 113 | Length of stay in the community/Year of residency | ________________years | |
| 114 | Owning a source of media | 1. No<br>2. TV<br>3. Radio<br>4. Mobile phone<br>5. Others___________ | |

PART- B: ***WATER, SANITATION AND HYGIENE CHARACTERISTICS***

| Section I: HOUSEHOLD QUESTIONNAIRE | | | |
|---|---|---|---|
| SR.No. | Questions | Coding Categories | Skip |
| 115 | The main source of drinking water for members household | 1 Piped into dwelling<br>2 Piped to yard/plot<br>3 Piped to neighbor<br>4 Public tap/standpipe<br>5 Tube well or Borehole<br>6 Protected well<br>7 Unprotected well<br>8 Protected spring<br>9 Unprotected spring<br>10 Rainwater<br>11 Tanker truck or ‘boti’<br>12 Cart with small<br>13 Surface water (river/dam/<br>14 Lake/pond/stream/canal/<br>15 Irrigation channel)<br>16 Bottled water<br>17 Other specify___________ | |
| 116 | Time take to fetch drinking water from the source<br>NB: ask for the time required for both trip including waiting time? | Rainy season _______minutes<br><br>Dry season _______minutes | |
| 117 | Do you do anything to the water to make it safer to drink? | 1 Yes<br>2 No | if 1 skip to Q118 |

| | | | |
|---|---|---|---|
| | | | if 2 skip to Q119 |
| 118 | What do you usually do to make the water safer to drink? | 1 Boil<br>2 Add bleach/chlorine<br>3 Strain through a cloth<br>4 Use a water filter (ceramic/<br>5 Sand/composite<br>6 Solar disinfection<br>7 Let it stand and settle<br>8 Other_____________ | |
| 119 | Is drinking water kept in a separate container, have a narrow mouth/opening, have a lid/cover, taken by pouring not dipping and water container clean both inside and outside? (Observe)<br>NB: If it misses one from above mentioned criteria the answer will be No | 1. Yes<br>2. No | |
| **Section- II: OBSERVATION**<br>The following questions are based on observation of the latrine. So the enumerator requests the respondent for observation. | | | |
| 120 | What kind of toilet facility do members of your household usually have? (Observe) | 1 Flush to the piped sewer system<br>2 Flush to a septic tank<br>3 Flush to a pit latrine<br>4 Flush to somewhere else<br>5 Flush, don't know where<br>6 Ventilated improved pit latrine<br>7 Pit latrine with slab<br>8 Pit latrine without slab/open pit<br>9 Composting toilet<br>10 Bucket toilet<br>11 Hanging toilet/hanging latrine<br>12 No facility/bush/field<br>13 Other _____________ | |
| 121 | The distance of toilet facility from dwelling house (count) | _____________ footstep | |
| 122 | The existing status of the toilet (Observe) | 1 Needs maintenance/Broken<br>2 No need maintenance | |
| 123 | What material the slab is made of? (Observe) | 1. Concrete / plastered and easy to clean<br>2. Mud and easy to clean | |

| | | | |
|---|---|---|---|
| | | 3. Concrete / plastered or mud but not easy to clean<br>4. Logs, stones (may be with sum mud), difficult to clean | |
| 124 | Is there feces on the slab of toilet? (Observe) | 1. Yes<br>2. No | |
| 125 | Is there feces on the wall of toilet? (Observe) | 1. Yes<br>2. No | |
| 126 | Is there flies entering and leaving the toilet? (Observe) | 1. Yes<br>2. No | |
| 127 | Do the toilet have offensive odor? (smell) | 1. Yes<br>2. No | |
| 128 | Do the toilet drop hole have the cover? (Observe) | 1. Yes<br>2. No | |
| 129 | Do the toilet Ensuring privacy? (Observe) | 1. Yes<br>2. No | |
| 130 | Cleanliness of the surrounding environment (Observe) | 1. Yes<br>2. No | |
| 131 | Do households utilize latrine? (observe)<br>NB: latrine is in use when: all the family members (above 5 years) use the latrine, clear pathway to the toilet on the ground, presence of anal cleansing materials, wet slab, feces in the pit. | 1. Yes<br>2. No | |
| 132 | When at home or nearby, how often do members of your household use latrine for defecation. (Observe) | 1. Always<br>2. Mostly<br>3. Sometimes<br>4. Rarely | |
| 133 | Open defecation surrounding house/compound (observe) | 1. Yes<br>2. No | |
| 134 | Do handwashing facility fixed near latrine? (observe) | 1. Yes<br>2. No | |
| 135 | Is there any sort of ashes or soap with handwashing facility? (observe) | 1. Yes<br>2. No | |
| 136 | Do households practice hand washing? (observe)<br>NB: you will see mark of water path and wetness on the ground around handwashing facility. | 1. Yes<br>2. No | |
| 137 | Do you have any space or area for toilet construction? (Observe) | 1. Yes<br>2. No | |
| 138 | When your latrine does constructed (year of construction)? | ______________________E.C. | |
| 139 | Do you share this toilet facility with other households? | 1. Yes<br>2. No | |
| 140 | How the sludge is treated if the pit gets full? | 1. Plant tree on it<br>2. I compost it<br>3. I use it as fuel | |

| | | | |
|---|---|---|---|
| | | 4. Cover it and dig another pit<br>5. Other specify______________ | |
| 141 | After what time you plan to upgrade this facility into an improved toilet such as Composting or Biogas? | _____________ | |
| 142 | What kind of sanitation approach promoted here?<br>NB: The enumerator have to identify the approach by asking the respondents how he/she constructed the toilet for the first time? Or who told them to do so? | 1. CLTS program<br>2. PHAST program<br>3. Sanitation subsidy<br>4. Through political leaders directives<br>5. School WASH program<br>6. Health extension Program<br>7. Other specify______________ | |
| 143 | Since when have you been promoted by this program?<br>NB: When does they started to tell you to do so? Still they are doing so? | Since ______to___________ | |
| 144 | Does poor sanitation cause disease? | 1. Yes<br>2. No | if 1 skip to Q145 |
| 145 | Could you name diseases caused by poor sanitation conditions (at least 3)? | 1. ___________<br>2. ____________<br>3. ____________<br>4. ____________<br>5. _____________<br>6. Do not Know | |
| 146 | Did your under 5 year children had diarrhea in the last 2 weeks? | 1. Yes<br>2. No | |
| | Could you name preventive measures (at least 3) of diarrhea? | 1. ___________________<br>2. ____________________<br>3. ____________________<br>4. ____________________<br>5. ___________________<br>6. Do not Know | |
| 147 | Do you have a family member with a chronic disease? such as;<br>• chronic joint problems,<br>• heart, and circulatory conditions,<br>• cancer,<br>• diabetes,<br>• chronic pulmonary diseases, or<br>• psycho-illness | 7. Yes<br>8. No | |

PART- *C:* ***AGRICULTURE AND LIVESTOCK CHARACTERISTICS***

| PART C: HOUSEHOLD QUESTIONNAIRE | | | |
|---|---|---|---|
| SR.No. | Questions | Coding Categories | Skip |

| | | | |
|---|---|---|---|
| 148 | Do you have farmland or Co-investing farmland? | 1 Yes<br>2 No | |
| 149 | Size of land owned by the household? | ______________Timad | |
| 150 | Do you use inorganic fertilizer (Urea, DAP and others) | 1 Yes<br>2 No | |
| 151 | How much amount of inorganic fertilizer can you afford | ______________ quintal | |
| 152 | How much amount of inorganic fertilizer couldn't you afford | _______________quintal | |
| 153 | Is there domestic animals in the house hold? | 1. Yes<br>2. No | |
| 154 | Number of domestic animals | 1. Cattle_________<br>2. Sheep________<br>3. Goat___________<br>4. Donkey______<br>5. Horse_______<br>6. Mule_____<br>7. Hen ___________<br>8. Dog___________<br>9. Cat____________ | |
| 155 | Do you use manure as fertilizer? (Observe) | 1. Yes<br>2. No | |
| 156 | Whose domestic animal manure do you use for fertilizer or you thought to be used? | 1. Cattle<br>2. Sheep<br>3. Goat<br>4. Donkey<br>5. Horse<br>6. Mule<br>7. Hen<br>8. Dog<br>9. Cat | |
| 157 | How much manure do use this year? | _______________quintal | |
| 158 | How much amount of manure couldn't you afford | _______________quintal | |
| 159 | What other thing did you use as fertilizer? | _______________________. | |
| 160 | Is there animal manure disposed here and there or piled in the compound other than compost? (Observe) | 1. Yes<br>2. No | |
| 161 | What type of energy do you use for cooking or preparing food? | 1. Electricity<br>2. Firewood<br>3. wood coal<br>4. Dried cow dung<br>5. Crop stalk<br>6. Other ___________ | |
| 162 | What do you use the source of light during night? | 1. Using Kerosene "kuraz"<br>2. Electricity<br>3. Generator | |

| | | | |
|---|---|---|---|
| | | 4. Solar system<br>5. Flash light<br>6. Other________ | |

PART- D: USER PERCEPTION WITH SANITATION FACILITY

Please indicate how much you satisfied and how much you belief on the following question

Please tick (√) on one cell you choose.

| Sr. No. | Question | Very satisfied | Somewhat satisfied | Less than satisfied | Completely dissatisfied |
|---|---|---|---|---|---|
| 163 | How satisfied are you with your main defecation facility?' | | | | |

Please indicate what you perceive on the following question Please tick (√) on one cell you choose.

| Sr. No. | Question | Very clean | Clean | Dirty | Very dirty |
|---|---|---|---|---|---|
| 164 | What do you feel about the cleanliness of the main defecation facility?' | | | | |

**NB**: Additional questions for the female member of the household regarding their perceptions of safety, privacy, and harassment Please indicate what you feel on the following question Please tick (√) on one cell you choose

| Sr. No. | Question | At night | During day time | Both night and day time | Not at both time |
|---|---|---|---|---|---|
| 165 | When do you feel safe to use the main defecation facility?' | | | | |

PART- E: WILLINGNESS OF HOUSEHOLDS TO PAY (WTP) IMPROVED SANITATION TECHNOLOGY THROUGH A USER-PAY APPROACH

**INSTRUCTION**: The questionnaire bellow will be answered by circling or writing figures.

| SR.No. | Questions | Coding Categories | Skip |
|---|---|---|---|
| 166 | The cost of constructing an Ecological toilet is 50,000 ETB, are you willing to pay that amount? | 1. Yes<br>2. No<br>3. Do not Know | If Yes go to Q167; if No go to Q169; If Don't know go to Q171. |
| 167 | What if the cost is 62,500 ETB, will you be willing to pay? | 1. Yes<br>2. No<br>3. Do not Know | If Yes go to Q168; If No Stop; If Don't know go to Q171. |
| 168 | What if the cost is 75,000 ETB, will you be willing to pay? | 1. Yes<br>2. No<br>3. Do not Know | If Yes go to Q171; If No Stop; If Don't know go to Q171. |
| 169 | What if the cost is 37,500 ETB, will you be willing to pay? | 1. Yes<br>2. No<br>3. Do not Know | If Yes go to Q171; If No go to Q170; If Don't know go to Q171. |
| 170 | What if the cost is 25,000 ETB, will you be willing to pay? | 1. Yes<br>2. No<br>3. Do not Know | If Yes stop; If No go to Q171; If Don't know go to Q171. |
| 171 | What is the maximum amount you are willing to pay for the construction of a Ecological toilet? | ___________________ETB | |

## PART-F: WILLINGNESS OF HOUSEHOLD TO USE HUMAN EXCREMENT

A. Statements on perceptions and attitudes of the households regarding human excreta

Please specify how much you agree or disagree with each of the following statements please tick (√) on the answer

| Sr. No. | Statement | Scores | | | | |
|---|---|---|---|---|---|---|
| | | Strongly agree | Agree | Disagree, | Strongly disagree | Don't know |
| 171 | Human excreta is a waste and suitable only for disposal | | | | | |
| 172 | Handling excreta is a great health risk | | | | | |
| 173 | Human excreta should not be handled in any way | | | | | |
| 174 | Human urine has no benefit to humans | | | | | |
| 175 | It is a taboo to handle urine | | | | | |
| 176 | Human faeces have no benefit to humans | | | | | |
| 177 | It is a taboo to touch faeces | | | | | |
| 178 | It is a taboo to touch treated faeces | | | | | |

PART-F: WILLINGNESS OF HOUSEHOLD TO USE HUMAN EXCREMENT

B. Statement on awareness of residents about the use of human excreta as fertilizer

Please tick (√) on answer how much you agree or disagree on each of the following statements

| Sr. No. | Statement | Scores | | |
|---|---|---|---|---|
| | | Agree | Do not Know | Disagree |
| 179 | Human excreta are a resource for the soil | | | |
| 180 | Sanitized human excreta can be used as fertilizer | | | |
| 181 | I will use human excreta on my crops if sanitized | | | |
| 182 | Taste of vegetables will change when fertilized with urine | | | |
| 183 | The smell of vegetables will change when fertilized with urine | | | |
| 184 | Crops can be killed when fertilized with urine | | | |
| 185 | Crops fertilized with human excreta are good for consumption | | | |
| 186 | I will never consume crops fertilized with human excreta | | | |
| 187 | Animal manure can be used as fertilizer | | | |
| 188 | Ever used animal manure as fertilizer | | | |

PART-F: WILLINGNESS OF HOUSEHOLD TO USE HUMAN EXCREMENT

C. The reason limiting the use of Eco-Toilets System (ETS) or treated excreta on their crops

**INSTRUCTION**: The questionnaire bellow will be answered by circling or writing figures.

| SR.No. | Questions | Coding Categories | Skip |
|---|---|---|---|
| 189 | Why did not you use treated feces on your crop? | 1. I don’t need it (enough animal manure available)"<br>2. Smell<br>3. Health risk<br>4. Appearance<br>5. Patronage will be poor<br>6. People will mock at me<br>7. Religious belief<br>8. Other_____________________ | |
| 190 | Why did not you use treated urine on your crop? | 1. I don’t need it (enough animal manure available)"<br>2. Smell<br>3. Health risk<br>4. Appearance<br>5. Patronage will be poor<br>6. People will mock at me<br>7. Religious belief<br>8. Other_____________________ | |
| 191 | What factors would encourage you to install the ETS at your home? | 1. Free Installation<br>2. Conserve water<br>3. Nutrient reuse<br>4. Others_____________ | |
| 192 | What factors would discourage you to install the ETS at your home? | 1. Technology not tested<br>2. Expensive<br>3. Complicated<br>4. Others________________ | |

PART-F: WILLINGNESS OF HOUSEHOLD TO USE HUMAN EXCREMENT

D. Statements to evaluate the predictors of the behavioral intention of Eco-toilet use of the Girar Jarso community

To respond to this section please tick (√) on one answer of the following listed items on a five-point scale statement.

| Sr. No. | Statement | Strong Disagreement | Moderate Disagreement | Neutral or undecided | Moderate Agreement | Strong Agreement | **Constructs** |
|---|---|---|---|---|---|---|---|
| 193 | The community leaders will support the IOM of the ETS | | | | | | Political |
| 194 | The local government will support the IOM of the ETS | | | | | | Political |
| 195 | The solution to water quality problems is a priority of our community leaders | | | | | | Political |
| 196 | Using the system is necessary for my lifestyle | | | | | | User demand |
| 197 | The ETS will motivate us to improve the health in the community | | | | | | User demand |
| 198 | I understand that the ETS has economic benefits | | | | | | User demand |
| 199 | The ETS will help solve the water-borne disease problems in my community | | | | | | User demand |
| 200 | We need more toilet in the community | | | | | | User demand |
| 201 | I hesitate to use the ETS for fear of making mistakes I cannot correct | | | | | | Anxiety |
| 202 | I am afraid I cannot operate and maintain the ETS well | | | | | | Anxiety |
| 203 | I am afraid that the ETS might be unsanitary | | | | | | Anxiety |
| 204 | I feel apprehensive and discomfort about using the ETS | | | | | | Anxiety |
| 205 | The ETS is somewhat intimidating to me | | | | | | Anxiety |
| 206 | My interaction with the ETS would be clear and understandable | | | | | | Ease of use |
| 207 | It is easy for me to learn and become skillful at using the ETS | | | | | | Ease of use |
| 208 | I found ETS; useful and efficient in improving the sanitation of my community | | | | | | usefulness |
| 209 | Using the ETS will enable me to improve the sanitation in my community faster | | | | | | usefulness |
| 210 | Using the ETS would motivate me to improve the sanitation in my community | | | | | | usefulness |
| 211 | Using the ETS is a good idea | | | | | | usefulness |
| 212 | Using the ETS is can be a source of income generation | | | | | | usefulness |
| 213 | The ETS makes my lifestyle more interesting | | | | | | Attitude towards |
| 214 | I like to use the ETS | | | | | | Attitude towards |
| 215 | I have the resource necessary to use the ETS (Money, space, etc.) | | | | | | Control behavior |
| 216 | I know the benefit of using the ETS | | | | | | Control behavior |
| 217 | A specific body is available for assistance in case of difficulties using the ETS. | | | | | | Control behavior |
| 218 | People who are important to me would think that using the ETS is good for me | | | | | | Subjective norm |
| 219 | People who influence my behavior would think that I should use the ETS | | | | | | Subjective norm |
| 220 | In general, my community has supported the use of the ETS | | | | | | Subjective norm |
| 221 | I plan to use the ETS in the next 12 months | | | | | | Intention to use |
| 222 | I have no plan to use the system in the next 12 months. | | | | | | Intention to use |

**NB**: IOM = implementation Operation and Maintenance; ETS = Eco-Toilets System

PART-G: ENVIRONMENTAL BELIEFS OF HOUSEHOLD

A. Statements to evaluate the predictors of the environmental beliefs of the Girar Jarso community

To respond to this section please tick (√) on one answer of the following listed items on a five-point scale statement.

| Sr. No. | Statement | Strongly agree | Agree | Disagree, | Strongly disagree | Don't know |
|---|---|---|---|---|---|---|
| 223 | "Do you think that natural resources in your community has gradually been depleted?" | | | | | |
| 224 | "According to you, to what extent has human behaviour contributed to the depletion of natural resources in your community?" | | | | | |
| 225 | "According to you, to what extent has climate change (global warming, more frequent extreme weather events) has contributed to the depletion of natural resources in your community?" | | | | | |
| 226 | "According to you, to what extent has incorrect institutional governance has contributed to the depletion of natural resources in your community?" | | | | | |
| 227 | "According to you, to what extent has increasing population has contributed to the depletion of natural resources in your community?" | | | | | |
