# Supplementary material for: Barriers to transition to resource-oriented sanitation in rural Ethiopia
Source: Environ Sci Pollut Res Int. 2025 Jan 14;32(5):2668–81. doi: 10.1007/s11356-025-35887-6 (PMC11802593; doi:10.1007/s11356-025-35887-6)
Supplement: Supplementary file 2 — Supplementary Information 2 (DOCX 403 KB) [file 11356_2025_35887_MOESM2_ESM.docx]

**SUPPLEMENTARY INFORMATION**

Table S1: Latent construct construction and distribution of responses for specific items (shares of respondents)

| Latent construct | Item specification | Strongly disagree | Disagree | Neutral or undecided | Agree | Strongly agree |
| --- | --- | --- | --- | --- | --- | --- |
| Perceived usefulness | I found ROTS useful and efficient in improving the sanitation of my community | 0.20 | 0.30 | 0.11 | 0.30 | 0.09 |
|  | Using the ROTS would motivate me to improve the sanitation in my community | 0.25 | 0.30 | 0.12 | 0.23 | 0.10 |
| Perceived ease of use | My interaction with the ROTS would be clear and understandable | 0.06 | 0.15 | 0.12 | 0.49 | 0.18 |
|  | It is easy for me to learn and become skilful at using the ROTS | 0.06 | 0.15 | 0.09 | 0.49 | 0.21 |
| Attitudes | Using the ROTS is a good idea | 0.04 | 0.09 | 0.08 | 0.51 | 0.28 |
|  | Using the ROTS can be a source of income generation | 0.02 | 0.10 | 0.10 | 0.51 | 0.27 |
|  | The ROTS can make my lifestyle more interesting | 0.04 | 0.10 | 0.10 | 0.49 | 0.27 |
|  | I like to use the ROTS | 0.07 | 0.09 | 0.08 | 0.34 | 0.42 |
| Social norms | People who are important to me would think that using the ROTS is good for me | 0.25 | 0.31 | 0.13 | 0.22 | 0.09 |
|  | People who influence my behaviour would think that I should use the ROTS | 0.28 | 0.31 | 0.14 | 0.22 | 0.05 |
|  | In general, my community has supported the use of the ROTS | 0.25 | 0.33 | 0.15 | 0.20 | 0.07 |
| Perceived control | I have the resource necessary to use the ROTS (Money, space, etc.) | 0.31 | 0.24 | 0.13 | 0.23 | 0.09 |
|  | I know the benefit of using the ROTS | 0.14 | 0.25 | 0.20 | 0.29 | 0.12 |
|  | A specific body is available for assistance in case of difficulties using the ROTS. | 0.27 | 0.35 | 0.16 | 0.18 | 0.04 |
| Perceived political support | The community leaders will support the IOM of the ROTS | 0.31 | 0.20 | 0.05 | 0.28 | 0.16 |
|  | The local government will support the IOM of the ROTS | 0.30 | 0.18 | 0.05 | 0.35 | 0.12 |
| Excreta use knowledge | Human excreta are a resource for the soil* |  | 0.08 | 0.12 | 0.80 |  |
|  | Sanitized human excreta can be used as fertilizer* |  | 0.10 | 0.15 | 0.75 |  |
|  | Crops fertilized with human excreta are good for consumption* |  | 0.17 | 0.22 | 0.62 |  |
| Excreta handling anxiety | Handling excreta is a great health risk | 0.07 | 0.09 | 0.02 | 0.35 | 0.47 |
|  | Human excreta should not be handled in any way | 0.08 | 0.22 | 0.04 | 0.25 | 0.41 |
|  | Human urine has no benefit to humans | 0.17 | 0.29 | 0.07 | 0.24 | 0.23 |
|  | It is a taboo to handle urine | 0.12 | 0.12 | 0.05 | 0.32 | 0.39 |
|  | Human faeces have no benefit to humans | 0.20 | 0.29 | 0.07 | 0.17 | 0.27 |
|  | It is a taboo to touch faeces | 0.12 | 0.12 | 0.05 | 0.33 | 0.38 |
|  | It is a taboo to touch treated faeces | 0.14 | 0.16 | 0.05 | 0.28 | 0.37 |

Notes: *Measured on a 3-point scale: Disagree – Don’t know – Agree; ROTS – resource-oriented toilet system

Table S2: Descriptive characteristics of respondents and their households

| Variables used in the analysis: | Statistics |
| --- | --- |
| Age of household head in years, mean (SD) | 45 (13) |
| Sex of household head (share of households with male heads) | 0.63 |
| Size of household, mean (SD) | 5.03 (2.04) |
| Education of household head (shares of: illiterate – can write and read – primary school – secondary school – college and above | 0.57 – 0.22 – 0.13 – 0.06 – 0.02 |
| Household income – annual, both in-cash and in-kind, from various sources in Ethiopian Birrs, mean (SD); values transformed by square root used in the analysis | 22653 (19536) |
| Farmland size in timads (SD) | 6.44 (6.17) |
| Livestock ownership in tropical livestock units (weights: large cattle 0.8; smaller cattle 0.6; sheep and goats 0.1; donkeys 0.4; hens and chickens 0.01), (SD) | 4.53 (2.57) |
| Shares of households having mobile phones – TVs – radios (used for the construction of media sources availability index by factor analysis) | 0.44 – 0.15 – 0.40 |
| Other descriptive characteristics of the sample | Statistics |
| Share of households with children below 10 years | 0.91 |
| Share of households with people above 60 years | 0.30 |
| Marital status of household head: shares of married – single – widowed – divorced | 0.84 – 0.07 – 0.06 – 0.03 |
| Occupation of household head – share of farmers | 0.89 |
| Ethnicity – share of Oromo | 0.99 |
| Religion – share of Ethiopian Orthodox | 0.97 |

Figure S1: Estimates for the model informed by TAM


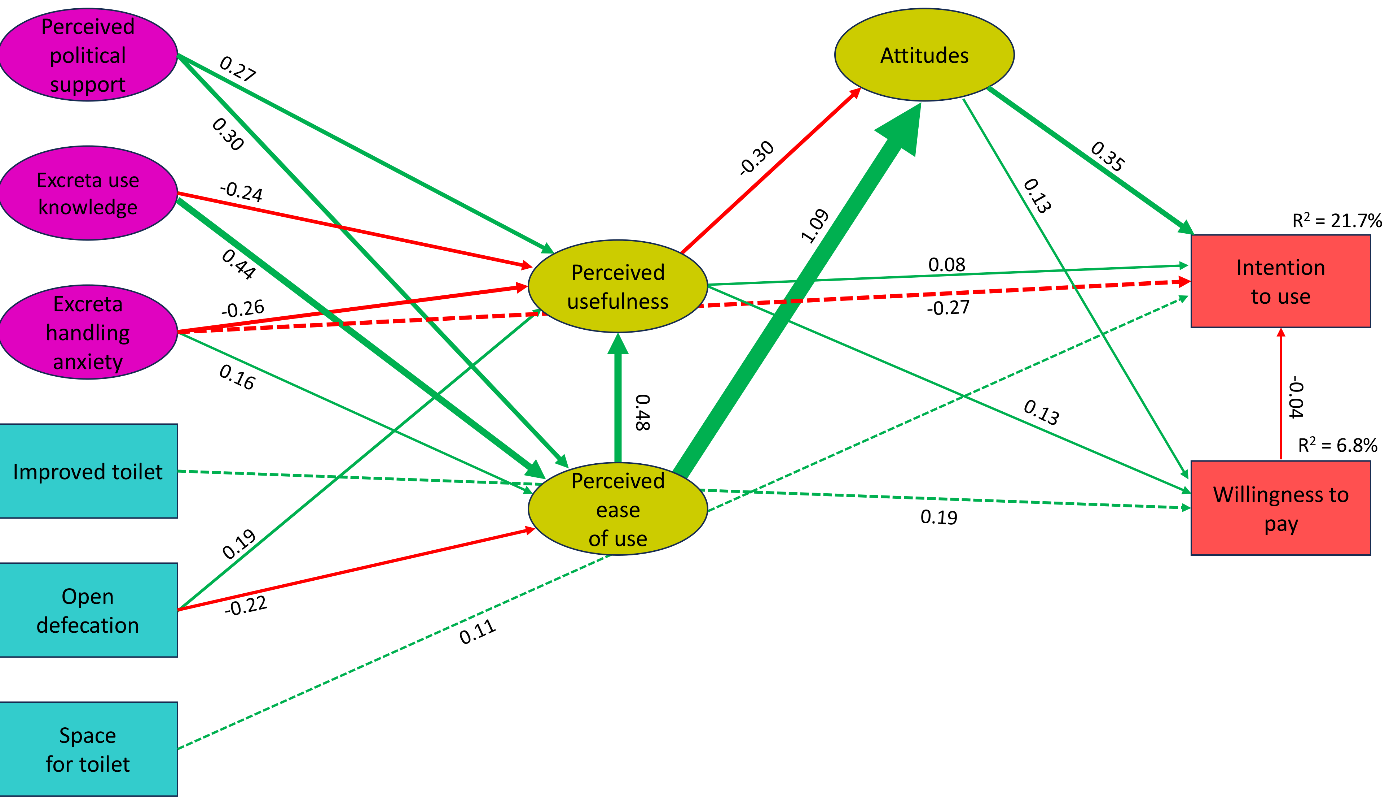


Figure S2: Estimates for the model informed by C-TAM-TPB


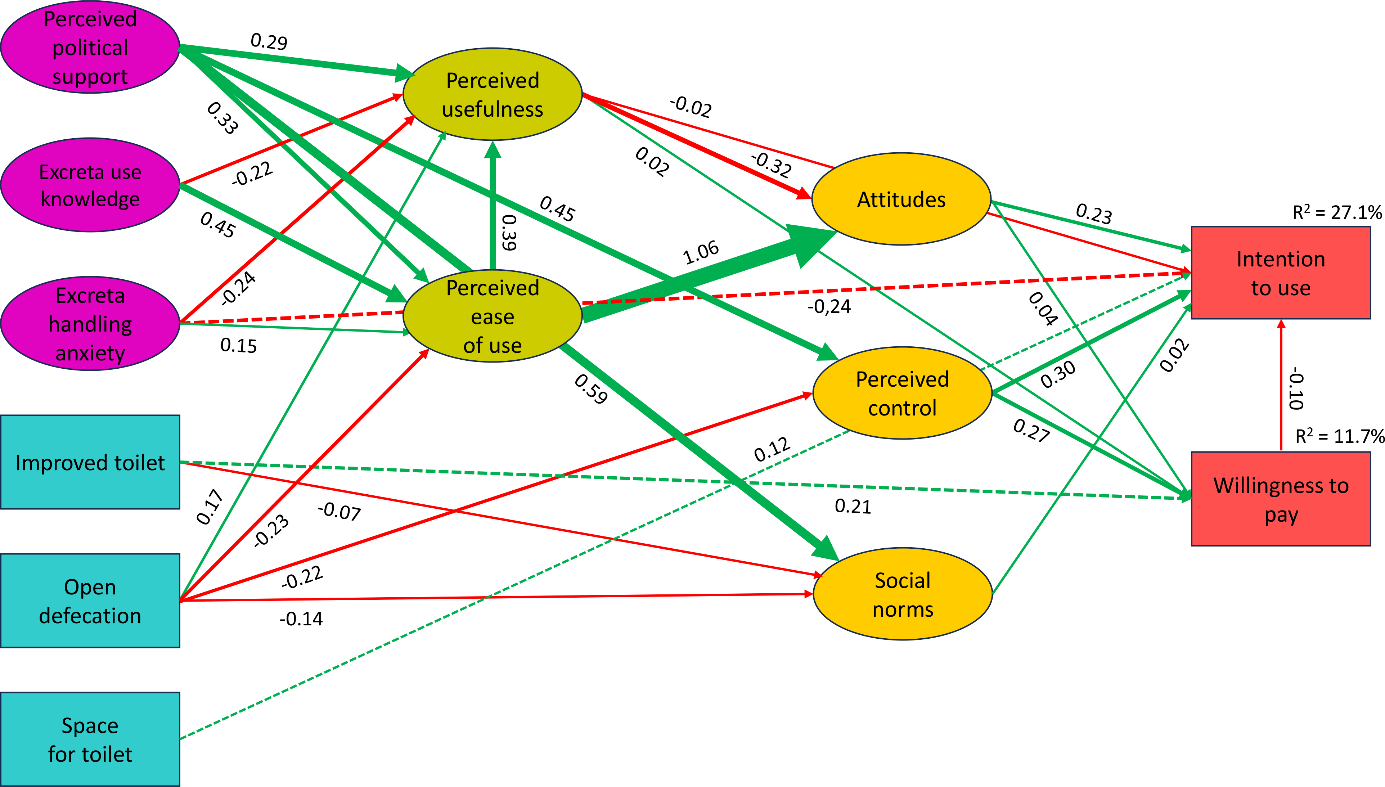


Table S3: Standardized direct, indirect, and total effects on intention to use ROTS

|  | Direct effect | | | Indirect effect | | | Total effect | | |
| --- | --- | --- | --- | --- | --- | --- | --- | --- | --- |
|  | TAM | TPB | C-TAM-TPB | TAM | TPB | C-TAM-TPB | TAM | TPB | C-TAM-TPB |
| Perceived ease of use | . | . | . | 0.368 | . | 0.199 | 0.368 | . | 0.199 |
| Perceived usefulness | 0.082 | . | -0.025 | -0.109 | . | -0.074 | -0.027 | . | -0.099 |
| Attitudes | 0.352 | 0.194 | 0.228 | -0.003 | -0.001 | -0.003 | 0.349 | 0.193 | 0.225 |
| Perceived control | . | 0.349 | 0.295 | . | -0.026 | -0.021 | . | 0.323 | 0.274 |
| Social norms | . | -0.013 | 0.025 | . | . | . | . | -0.013 | 0.025 |
| Perceived political support | . | . | . | 0.102 | 0.174 | 0.176 | 0.102 | 0.174 | 0.176 |
| Excreta use knowledge | . | . | . | 0.168 | 0.098 | 0.111 | 0.168 | 0.098 | 0.111 |
| Excreta handling anxiety | -0.269 | -0.232 | -0.243 | 0.065 | 0.041 | 0.055 | -0.204 | -0.191 | -0.188 |
| Improved toilet | . | . | . | -0.004 | -0.016 | -0.018 | -0.004 | -0.016 | -0.018 |
| Open defecation | . | . | . | -0.087 | -0.119 | -0.125 | -0.087 | -0.119 | -0.125 |
| Space for toilet | 0.114 | 0.117 | 0.118 | . | . | . | 0.114 | 0.117 | 0.018 |

Table S4: Standardized direct, indirect, and total effects on WTP for ROTS

|  | Direct effect | | | Indirect effect | | | Total effect | | |
| --- | --- | --- | --- | --- | --- | --- | --- | --- | --- |
|  | TAM | TPB | C-TAM-TPB | TAM | TPB | C-TAM-TPB | TAM | TPB | C-TAM-TPB |
| Perceived ease of use | . | . | . | 0.184 | . | 0.047 | 0.184 | . | 0.047 |
| Perceived usefulness | 0.126 | . | 0.023 | -0.040 | . | -0.013 | 0.086 | . | 0.010 |
| Attitudes | 0.131 | 0.016 | 0.041 | . | . | . | 0.131 | 0.016 | 0.041 |
| Perceived control | . | 0.306 | 0.275 | . | . | . | . | 0.306 | 0.275 |
| Perceived political support | . | . | . | 0.078 | 0.138 | 0.143 | 0.078 | 0.138 | 0.143 |
| Excreta use knowledge | . | . | . | 0.060 | 0.008 | 0.019 | 0.060 | 0.008 | 0.019 |
| Excreta handling anxiety | . | . | . | 0.006 | 0.003 | 0.005 | 0.006 | 0.003 | 0.005 |
| Improved toilet | 0.194 | 0.205 | 0.206 | . | . | . | 0.194 | 0.205 | 0.206 |
| Open defecation | . | . | . | -0.025 | -0.071 | -0.068 | -0.025 | -0.071 | -0.068 |
| Space for toilet | . | . | . | . | . | . | . | . | . |
